# α-Synuclein as CSF and Blood Biomarker of Dementia with Lewy Bodies

**DOI:** 10.1155/2012/437025

**Published:** 2012-09-26

**Authors:** Kensaku Kasuga, Masatoyo Nishizawa, Takeshi Ikeuchi

**Affiliations:** ^1^Department of Neurology, Brain Research Institute, Niigata University, Niigata 951-8585, Japan; ^2^Department of Neurosciences, University of California, San Diego, 9500 Gilman Drive, La Jolla, San Diego, CA 92093, USA

## Abstract

Dementia with Lewy bodies (DLB) is a common subtype of dementia in the elderly. DLB is neuropathologically characterized by the presence of Lewy bodies and Lewy neurites, both of which are composed of **α**-synuclein. Although **α**-synuclein was initially considered to be an exclusively intracellular protein, it has been found to be secreted into biological fluids. **α**-Synuclein in biological fluids such as cerebrospinal fluid (CSF) and blood has been discussed as a potential biomarker of DLB and **α**-synuclein-related disorders, because **α**-synuclein is characteristically accumulated in the brain of patients with these disorders. The **α**-synuclein level in CSF has been examined by several investigators, and the majority of studies have shown a reduction in CSF **α**-synuclein level in DLB and **α**-synuclein-related disorders. Discrepant findings of studies of plasma **α**-synuclein level in patients with DLB have been reported. Because the level of **α**-synuclein stored in red blood cells is considerably high, blood contamination and haemolysis during sample collection and processing should be considered as a confounding factor for quantification of **α**-synuclein. Here, the recent progress in the studies of **α**-synuclein as a biomarker of DLB and their potential clinical applications are reviewed.

## 1. Introduction

Dementia with Lewy bodies (DLB) is a common subtype of dementia and is reported to be the second most common neurodegenerative dementia after Alzheimer's disease (AD) in the elderly in several studies [1–3]. DLB is a progressive cognitive disorder characterized by fluctuating cognitive impairment, visual hallucination, and parkinsonism [4]. Diagnosis of DLB in patients with such characteristic clinical features would not be difficult by taking medical history and careful neurological examinations. However, it could be laborious to make an accurate diagnosis of DLB when patients have a substantial degree of concomitant AD pathology, which affects the clinical symptoms with lower rates of visual hallucinations and parkinsonism [5, 6]. Accurate clinical diagnosis of DLB is important because patients may benefit from cholinesterase inhibitors, which improve cognitive function and neuropsychiatric symptoms of DLB [7]. Furthermore, it should be noted that DLB patients are particularly sensitive to neuroleptic drugs [4, 8]. Recent intensive research has given hope for disease-modifying therapeutics for DLB to become a reality. The evaluation of such therapies largely depends on reliable diagnostic and prognostic biomarkers for early detection and monitoring of the stage of DLB. Candidates of such biomarkers are diverse; clinical biomarkers detected on the basis of olfactory function [9], myocardial ^123^I-metaiodobenzylguanidine (MIBG) scintigraphy [10], neuroimaging biomarkers detected by magnetic resonance imaging (MRI), single photon emission computed tomography (SPECT) [11], positron emission tomography (PET), and biochemical biomarkers. In this paper, fluid biomarkers, particularly *α*-synuclein in cerebrospinal fluid (CSF) and blood in patients with DLB and *α*-synuclein-related disorders were extensively reviewed.

## 2. Lewy Bodies and ***α***-Synuclein

A century ago, in 1912, Lewy originally described neuronal inclusions in the brain of patients with Parkinson's disease (PD). Seven years later, a Russian neuropathologist, Tretiakoff, named the inclusions as “corps de Lewy (Lewy bodies)” [12]. Since the discovery, Lewy bodies have been considered as intracytoplasmic, spherical, and eosinophilic neuronal inclusions in the substantia nigra of PD patients. In 1976, Kosaka et al. reported the detection of Lewy bodies in the cerebral cortex of an elderly patient with dementia [13]. After his detailed description of cortical Lewy bodies [14], many similar cases were subsequently reported. Because the characteristic pathological features of DLB and PD are Lewy bodies and Lewy neurites, these disorders are considered to belong to a continuum disorder, and the generic term Lewy body disease (LBD) was proposed [4, 15].

In 1997, Spillantini et al. found that *α*-synuclein is the main component of Lewy bodies in the brains of PD and DLB patients [16]. Only two months before this report, a mutation was identified in *SNCA*, which encodes *α*-synuclein in families with autosomal dominant PD [17]. A different *SNCA* mutation was subsequently reported in a family with DLB [18]. In addition, *SNCA* multiplication by duplication/triplication has been identified in a large family with phenotypes ranging from DLB to PD [19]. These neuropathological and genetic findings suggest that *α*-synuclein is essentially implicated in the pathogenesis of LBD including DLB and PD. Recent studies have suggested that the Lewy body pathology propagates throughout the brain via neuron-to-neuron transmission of *α*-synuclein aggregates [20]. It has been demonstrated that glial cytoplasmic inclusions, which are unique pathological inclusions found in brains of patients with multiple system atrophy (MSA), are also composed of *α*-synuclein [21], suggesting an unexpected link between MSA and LBD. These disorders with pathological accumulation of *α*-synuclein in brains are termed as *α*-synucleinopathies [22].

*α*-Synuclein is a 140-residue ubiquitous protein and is highly expressed in neuronal presynaptic terminals under physiological conditions. Although its physiological functions remain unclear, *α*-synuclein is implicated in synaptic vesicle trafficking particularly in the regulation of synaptic vesicle release [23] and stabilization of SNARE complexes [24]. Deposited *α*-synuclein in the brain with synucleinopathies is aberrantly phosphorylated at Ser129 [25]. It has been demonstrated that the C-terminal truncated form of *α*-synuclein promotes the aggregation of *α*-synuclein [26]. Because *α*-synuclein has no signal sequence, it was initially considered to be an exclusively intracellular protein. However, it has been shown that *α*-synuclein is secreted into biological fluids [27, 28].

## 3. ***α***-Synuclein in CSF

A year after Spillantini et al. reported that *α*-synuclein is the main component of Lewy bodies, Jakowec et al. examined whether *α*-synuclein is detectable in CSF [29]. They examined the expression of *α*-synuclein in CSF from PD patients and control subjects by Western blot analysis using an anti-*α*-synuclein specific antibody, but failed to detect *α*-synuclein in CSF [29]. Borghi et al. detected *α*-synuclein by a method combining immunoprecipitation with immunoblot analysis using two different anti-*α*-synuclein antibodies [30]. In their study, however, the intensity of the bands immunoreactive to the anti-*α*-synuclein antibodies showed no significant difference between patients with PD and control subjects [30]. El-Agnaf et al. also confirmed that *α*-synuclein is detectable in CSF by a similar methodology [27].

Subsequently, Tokuda et al. demonstrated using a new system for enzyme-linked immunosorbent assay (ELISA) that PD patients showed significantly lower *α*-synuclein levels in CSF than the control groups [31]. Since then, several studies to determine the total *α*-synuclein level in CSF have been carried out by ELISA or bead-based flow cytometric assay using various combinations of anti-*α*-synuclein antibodies (Tables 1 and 2). A significantly low level of *α*-synuclein in CSF from patients with LBD (PD and/or DLB) has been shown by independent studies [28, 31–38]. Our study revealed that total CSF *α*-synuclein level shows a significant positive correlation with the CSF A*β*42 level in DLB patients [32]. It has been suggested that a low CSF A*β*42 level may be related to amyloid-related pathology in the brains of DLB patients [39]. In addition, experimental studies have suggested that the A*β*42 species strongly enhances the accumulation of *α*-synuclein [40]. In the CSF from LBD patients, the level of A*β*42 shows a positive correlation with the activity of neprilysin, an enzyme that degrades A*β* [41]. Taken together, the reduction in *α*-synuclein and A*β*42 levels in CSF suggests the extent of Lewy body pathology and the co-occurrence of amyloid pathology, respectively, in the brain of DLB patients. Interestingly, patients with *SNCA* duplication who showed abundant Lewy body pathology in the brain [42] also show low levels of CSF *α*-synuclein [32]. Because the predominant source of *α*-synuclein in CSF is considered to be the central nervous system [43], the decrease in the level of CSF *α*-synuclein in DLB may reflect a dysfunction in the metabolism or clearance of *α*-synuclein in the brain, similarly to AD patients with A*β* accumulation in the brain showing a decrease in the level of A*β*42 in CSF.

In contrast, comparable levels of total *α*-synuclein in CSF between patients with LBD and control subjects have also been reported [45–50]. This discrepancy is probably not due to the misdiagnosis of LBD, because different studies using CSF samples derived from autopsy-confirmed patients showed significantly decreased or comparable *α*-synuclein levels in LBD patients [34, 50]. In addition to methodological differences in the quantification of *α*-synuclein, blood contamination of CSF during lumbar puncture should be taken into account when considering the discrepant results. The level of *α*-synuclein in blood, particularly that stored in red blood cells, is much higher than that in CSF [33, 53]. Haemolysis in the course of sample collection and processing should be considered as a confounding factor for quantification of *α*-synuclein level in CSF and blood. Other factors such as level fluctuations over time and drug treatment may have less effect on the level of *α*-synuclein in CSF. Although the level of A*β* in CSF fluctuates over time [54], the level of *α*-synuclein in CSF does not significantly change [52]. It is also reported that drugs such as L-dopa and dopamine agonists do not affect the level of *α*-synuclein in CSF [34, 35]. 

Several groups have recently conducted studies to detect the oligomeric forms of *α*-synuclein in CSF, because the oligomer species of *α*-synuclein are considered to be toxic and could enhance pathological accumulation of *α*-synuclein and disease propagation [55]. Tokuda et al. demonstrated that the levels of *α*-synuclein oligomers in CSF are significantly higher in patients with PD than in patients with progressive supranuclear palsy (PSP) or AD [44]. In their study, the level of total *α*-synuclein in the CSF from PD patients tends to decrease [44]. Increased level of *α*-synuclein oligomers in CSF from PD patients was also shown by other investigator [51]. Foulds et al. showed that the level of oligomers composed of phosphorylated *α*-synuclein is higher in the postmortem CSF from MSA patients than in that from with PD, DLB, or PSP patients [50]. Sierks et al. also showed a significant increase in the level of *α*-synuclein oligomers in postmortem CSF from patients with PD by electrochemical impedance spectroscopy [56]. These findings suggest a possibility that *α*-synuclein oligomer species are detectable in CSF and that their levels may increase in some patients with synucleinopathies. A potential concern is that the oligomeric forms of *α*-synuclein detected in their studies using different methods may be heterogeneous in size and toxicity; hence, further validation is still needed.

Correlation analysis of clinical parameters, such as minimental state examination (MMSE) and Hoehn Yahr scale scores with the total *α*-synuclein level in CSF, has shown inconsistent results. Tokuda et al. showed an inverse correlation between the total *α*-synuclein level in CSF and disease severity determined by the Hoehn Yahr scale [31]. A low *α*-synuclein level was reported to correlate with a low MMSE score of DLB patients [48]. These findings suggest that *α*-synuclein level in CSF may reflect the severity of pathological changes occurring in patients with LBD. This notion is supported by the findings of a study that the disease duration in patients with DLB is closely associated with a low *α*-synuclein level in CSF [46]. In contrast to these findings, other studies revealed no significant association of the *α*-synuclein level in CSF with MMSE score, gender, age at examination, or disease duration in DLB or AD patients [32]. Shi et al. examined whether CSF *α*-synuclein level correlates with dopaminergic dysfunction determined by PET in asymptomatic carriers with leucine-rich repeat kinase 2 (LRRK2) gene mutation [64]. They detected no significant correlations, indicating that CSF total *α*-synuclein level may not be a sensitive biomarker of the preclinical phase of PD.

## 4. ***α***-Synuclein in Blood

Several studies on the quantification of *α*-synuclein in blood have been carried out because drawing blood is much less invasive than lumbar puncture to obtain CSF from patients (Table 3). El-Agnaf et al. detected *α*-synuclein in plasma of patients with LBD by immunoprecipitation using an anti-*α*-synuclein antibody [27]. Subsequently, they found higher levels of *α*-synuclein oligomers in plasma from PD patients than in that from control subjects by ELISA [57]. A similar increase in *α*-synuclein level was observed in plasma from patients with PD and MSA [58, 59]. Lee et al. found that plasma *α*-synuclein level is higher in patients with PD than in those with MSA [58]. Duran et al. demonstrated that drugs such as L-dopa, dopamine agonists, and MAO/COMT inhibitors do not affect the plasma *α*-synuclein level in patients with PD [59]. The phosphorylated *α*-synuclein level in plasma quantified by ELISA as well as Western blot analysis is higher in patients with PD than in control subjects [60]. In their study, the level of *α*-synuclein remained stable within the same individuals at least over 3 months.

By contrast, Li et al. found a significantly decreased *α*-synuclein level in plasma from patients with PD by Western blot analysis, which detected only full-length monomeric *α*-synuclein [62]. Laske et al. also reported a similar decrease in serum *α*-synuclein level in DLB patients compared with AD patients and control subjects [63]. Comparable levels of plasma *α*-synuclein were found among patients with PD, AD, and control subjects in other studies [61].

## 5. Conclusions

Results of measurements of *α*-synuclein level in CSF and blood have been variable; hence, it is difficult to unequivocally conclude whether *α*-synuclein is a promising fluid biomarker of DLB and other *α*-synucleinopathies. More discriminating results for DLB patients could be obtained by examining specific *α*-synuclein species such as truncated, phosphorylated, and oligomeric species on the basis of their analogy to A*β*42 and phosphorylated tau species whose changes in levels are found to be reliable CSF biomarkers of AD. In addition, a multicenter study is required to validate the usefulness of *α*-synuclein as a biomarker by standardized methods of quantifying *α*-synuclein. Continuous efforts will be required to establish useful fluid biomarkers for early diagnosis of DLB and evaluation of disease-modifying therapeutics for DLB.

## Figures and Tables

**Table 1:** Studies on quantification of *α*-synuclein level in CSF of patients with DLB and other synucleinopathies.

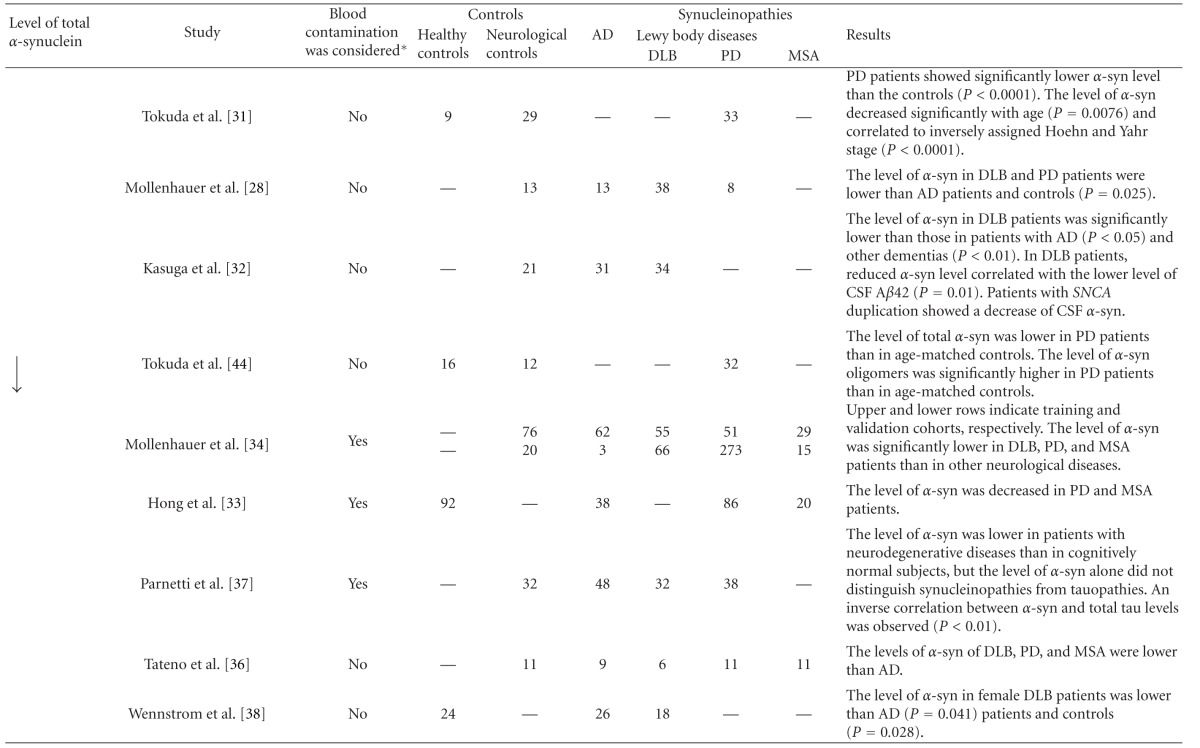 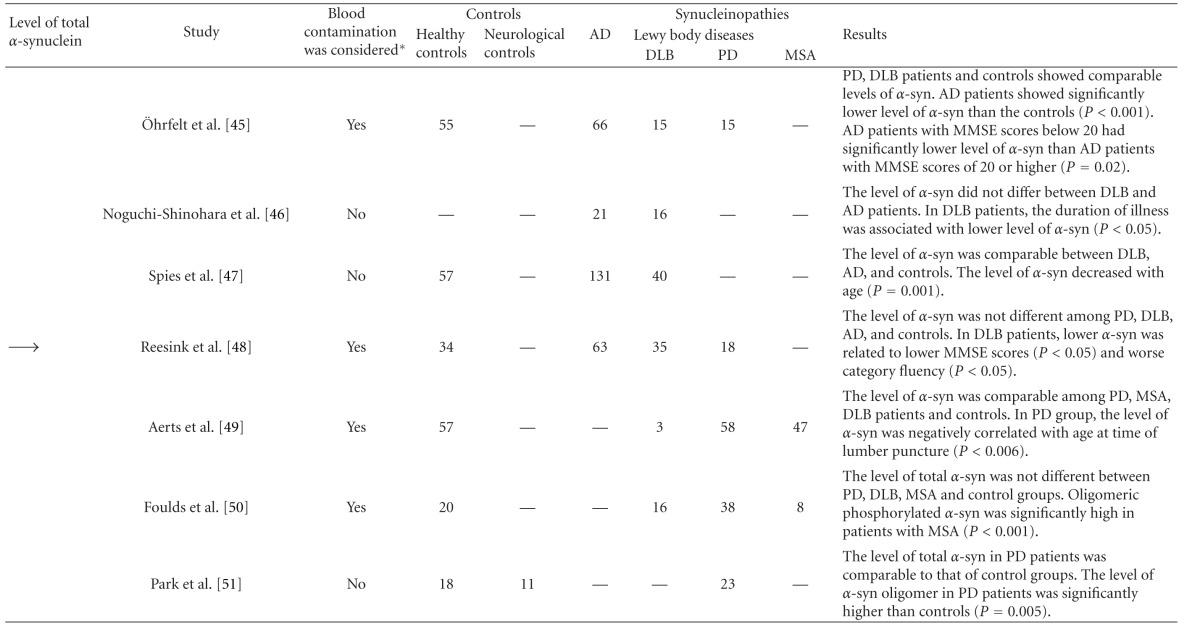

Arrows indicate decreased (↓) and comparable (→) levels *α*-synuclein. Sample numbers are shown in each category. *Erythrocyte counts or haemoglobin levels were considered as a confounding factor. AD: Alzheimer's disease; DLB: dementia with Lewy bodies; PD: Parkinson's disease; MSA: multiple system atrophy; *α*-syn: *α*-synuclein; MMSE: minimental state examination.

**Table 2:** Summary of antibodies used to quantify *α*-synuclein in biofluids.

Study	Target molecule	Anti-*α*-synuclein antibodies	
		Capture antibody	Detecting antibody
Tokuda et al. [31]	Total *α*-synuclein	211 (m)	FL-140 (p)
Mollenhouer et al. [28]	Total *α*-synuclein	mSA-1 (p)	Syn-1 (m)
Öhrfeltet al. [45]	Total *α*-synuclein	Syn1b (m)	Syn3b (m), Syn5d (m)
Noguchi-Shinohara et al. [46]	Total *α*-synuclein	211 (m)	FL-140 (p)
Spies et al. [52]	Total *α*-synuclein	211 (m)	FL-140 (p)
Kasuga et al. [32]	Total *α*-synuclein	Syn-1 (m)	FL-140 (p)
Reesink et al. [48]	Total *α*-synuclein	211 (m)	FL-140 (p)
Tokuda et al. [44]	Total *α*-synuclein	211 (m)	FL-140 (p)
	Oligomeric *α*-synuclein	211 (m)	Biotinylated 211 (m)
Aerts et al. [49]	Total *α*-synuclein	211 (m)	FL-140 (p)
Mollenhouer et al. [34]	Total *α*-synuclein	mSA-1 (p)	Syn-1 (m)
Hong et al. [33]	Total *α*-synuclein	211 (m), LB509 (m), rabbit anti-*α*-synuclein (p)	Biotinylated goat anti-human *α*-synuclein (p)
Parnetti et al. [37]	Total *α*-synuclein	211 (m)	FL-140 (p)
Foulds et al.[50]	Total *α*-synuclein	211 (m)	FL-140 (p)
	Oligomeric *α*-synuclein	211 (m)	Biotinylated 211 (m)
	Phosphorylated *α*-synuclein	N-19 (p)	pS129 (m)
	Oligomeric phosphorylated *α*-synuclein	pS129 (m)	Biotinylated pS129 (m)
Tateno et al. [36]	Total *α*-synuclein	Not described	Not described
Wennstrom et al. [38]	Total *α*-synuclein	Commercial kit (Invitrogen)	
Park et al. [51]	Total *α*-synuclein	211 (m)	FL-140 (p)
	Oligomeric *α*-synuclein	211 (m)	Biotinylated 211 (m)

m: monoclonal antibody; p: polyclonal antibody.

**Table 3:** Studies of quantification of *α*-synuclein level in blood of patients with DLB and other synucleinopathies.

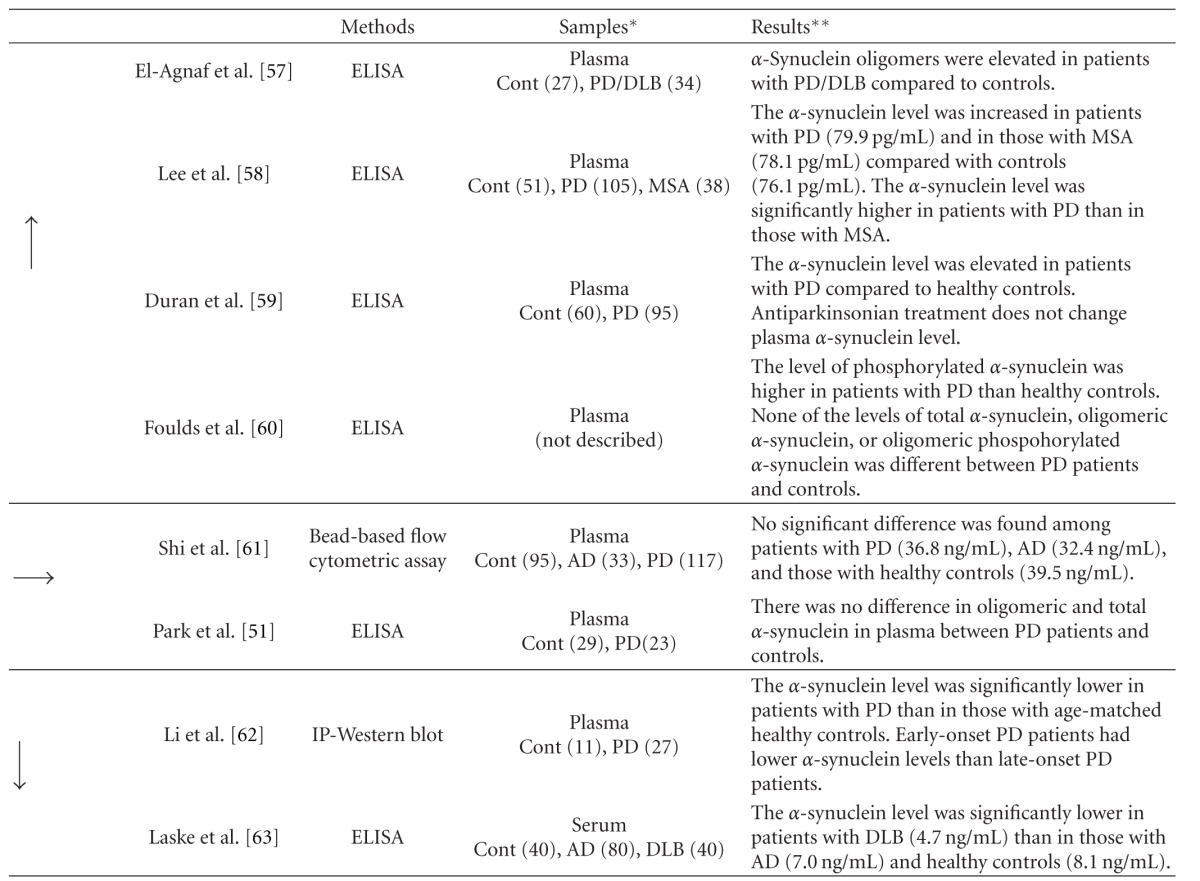

Arrows indicate increased (↑), comparable (→), and decreased (↓) levels of *α*-synuclein.*Sample numbers are shown in parenthesis. **Values are indicated as mean or median. AD: Alzheimer's disease; Cont: controls; DLB: dementia with Lewy bodies; IP: immunoprecipitation; MSA: multiple system atrophy; PD: Parkinson's disease.
